# Fish, mirrors, and a gradualist perspective on self-awareness

**DOI:** 10.1371/journal.pbio.3000112

**Published:** 2019-02-07

**Authors:** Frans B. M. de Waal

**Affiliations:** Living Links, Yerkes National Primate Research Center and Psychology Department, Emory University, Atlanta, Georgia, United States of America

## Abstract

The mirror mark test has encouraged a binary view of self-awareness according to which a few species possess this capacity whereas others do not. Given how evolution works, however, we need a more gradualist model of the various ways in which animals construe a self and respond to mirrors. The recent study on cleaner wrasses (*Labroides dimidiatus*) by Kohda and colleagues highlights this need by presenting results that, due to ambiguous behavior and the use of physically irritating marks, fall short of mirror self-recognition. The study suggests an intermediate level of mirror understanding, closer to that of monkeys than hominids.

## Abstract 2

In response to claims that fish recognize themselves in a mirror, hence are self-aware, this Primer argues that this conclusion is premature due to ambiguity of the observed responses and the methodology used; instead we need a gradualist approach to the evolution of self-awareness in animals

Complex cognitive capacities evolve bottom-up in small incremental steps from more basic traits shared across a wide range of species [1]. Therefore, we do not expect all-or-nothing cognitive differences between related species. Yet, for the capacity of self-awareness, we still live with a "Big Bang" theory, according to which this trait appeared out of the blue in just a handful of species, whereas the vast majority lacks it. This view has been with us for half a century, ever since Gallup [2] tested the responses of chimpanzees to mirrors. Without any specific training, anthropoid apes manually investigate a mark on their body that is visible only via a mirror, whereas rhesus macaques (and other monkeys) never do. This contrast within the primate order has prompted the assumption of a qualitative difference in self-concept that sets the hominids (humans and the great apes) apart. This contrast was later extended to other cognitive domains [3].

Challenges to this mental gap have been manifold and never-ending and cannot possibly all be reviewed here. An obvious method is to try to demonstrate mirror self-recognition (MSR) in nonhominids. Such attempts have been remarkably unsuccessful, however, except for a handful of species, notably bottlenose dolphins [4], Asian elephants [5], and Eurasian magpies [6]. Conversely, the mark test has failed to produce the required response in a great multitude of nonhominids, such as in a recent well-controlled study of large-brained *Psittaciformes* [7]. Alternatively, failure to find MSR in a given species has been attributed to lack of motivation (e.g., some animals may not care about paint on their bodies), trouble with attention (e.g., some animals avoid looking at "another” in the mirror), or a lack of perception (e.g., a visual paradigm may not suit an olfactory species), rather than the absence of a self-concept. There have also been attempts to explain away the mirror responses of apes, such as by attributing them to anesthesia ([8], countered by [9]). Others have trained animals to go through the motions indicative of a successful mark test, starting with conditioned pigeons [10]—a study that has proven impossible to replicate [11]—followed by extensively trained macaques [12].

Shaped by thousands of rewarded trials, mirror responses are about as meaningful as would be the literary talent of a monkey taught to type “to be or not to be.” (See [13] for a critique of these travesties of the original mirror test.) The only measure that counts is the untrained response to the first visual body mark detected with the assistance of a mirror. Speaking from first-hand experience, I have no doubt that chimpanzees treat a mirror differently than most animals. On a sunny day, it is common for them to use my sunglasses as mirrors. While staring into them, they inspect the inside of their mouth, opening it wide to feel their teeth with a finger while coordinating closely with their reflection. They may also turn around to inspect an injury on their back, or females will try to take a look at their genital swelling. This is why we hardly need a mark test to realize that apes connect their reflection with their own body (Fig 1). I have also extensively worked with monkeys yet never observed any spontaneous self-inspection in front of a mirror. Nevertheless, many non-MSR species, including monkeys, demonstrate a basic understanding of mirrors. They know how to use them as tools to see things that are otherwise invisible and distinguish their own reflection from a stranger (see below).

**Fig 1:**
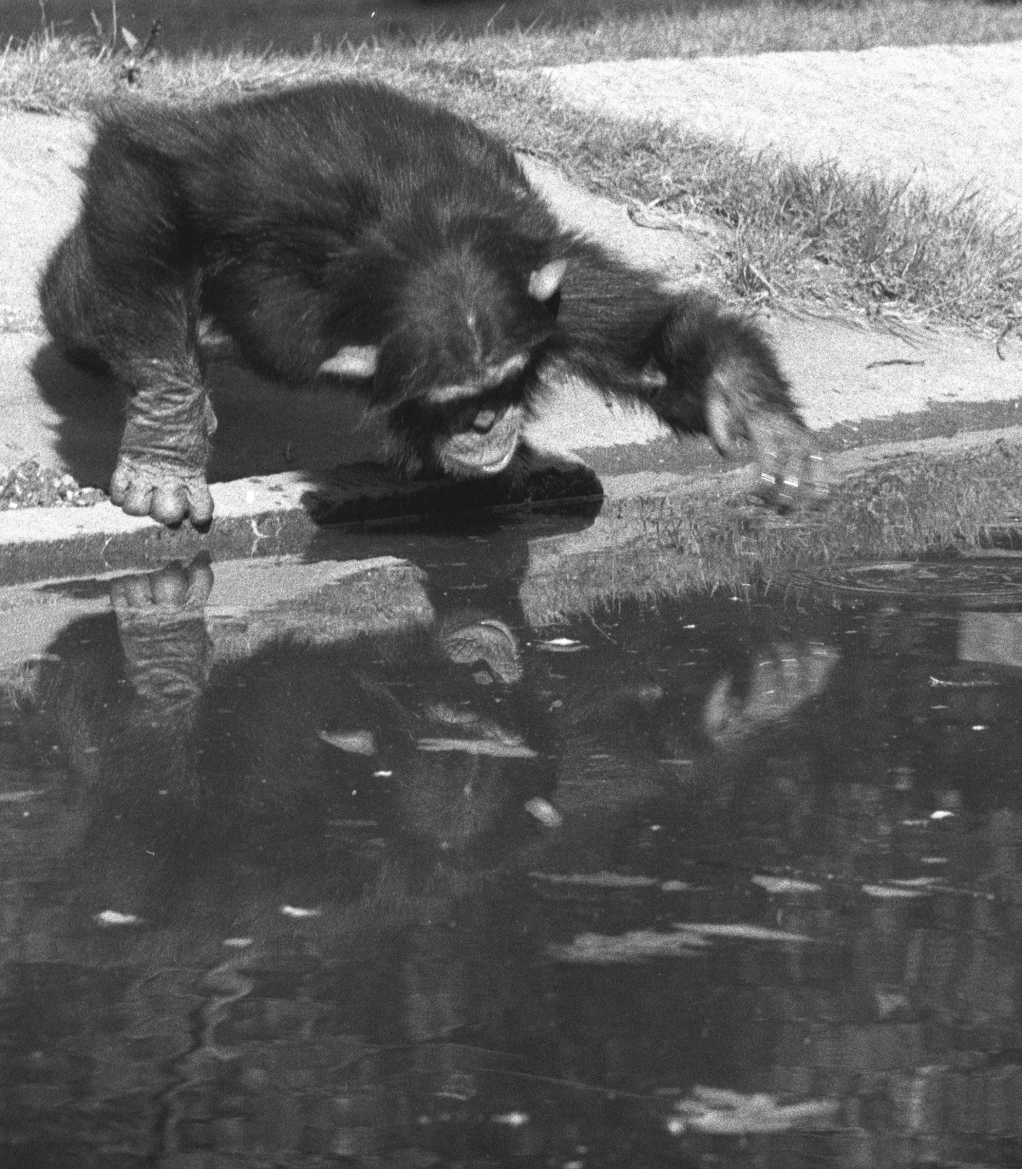
Chimpanzees spontaneously explore their mirror reflection by pulling strange faces or inspecting parts of their bodies that they cannot see otherwise. Here, a young male at a zoo stares at his own reflection in a water moat, occasionally disturbing the surface with his hand. Photograph by Frans de Waal.

MSR requires that the mirror test (a) be applied only when social reactions to the mirror have been replaced by self-directed behavior, such as testing the contingency between one’s own movements and those of one's reflection, (b) involve a purely visual mark, and (c) be done without previous training, least of all training of responses indicative of self-recognition. The most convincing MSR occurs in species capable of probing their own bodies, such as primates and elephants, or preening themselves at places they cannot see without a mirror, such as birds. Accordingly, one might think that only species with hands, trunks, or flexible necks can possess a self-concept. This rather absurd conclusion would follow from the mirror mark test and its reliance on self-touching and the visual sense, which explains why so many scientists have lamented its limitations.

This brings us to the current intriguing study by Kohda and colleagues [14] of cleaner wrasses, *Labroides dimidiatus*. This particular fish, which services larger host fish by cleaning them of dead skin and ectoparasites (Fig 2), is well known for its sophisticated social behavior and economic decision-making and is therefore not nearly as cognitively simple as *Osteichthyes* are typically assumed to be (e.g., [15]). The cleaner wrasse's spontaneous reactions to the mirror are hard to interpret, though. They include swimming upside down and repeats of 400 times per day of certain atypical behaviors in front of the mirror. However odd and unusual these movements may be, whether they amount to explorations of the contingency between the self and its reflection is as speculative as in another fish study in which giant manta rays stayed close to a mirror while performing repeated actions [16].

**Fig 2:**
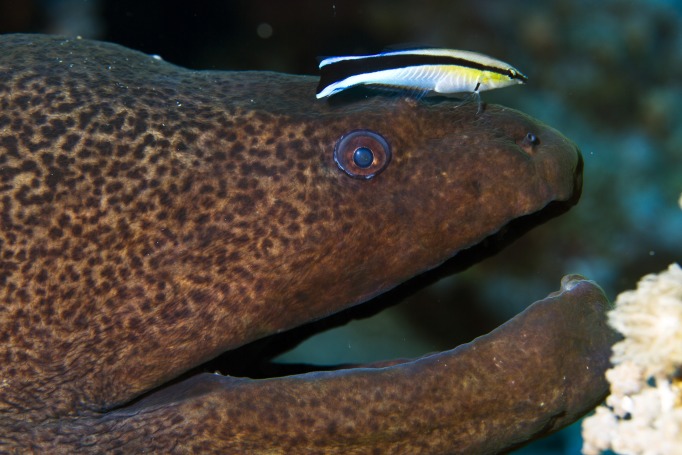
A cleaner wrasse, *Labroides dimidiatus*, attends to a giant moray eel, *Gymnothorax javanicus*. Image credit: Silke Baron (Flickr).

Generous interpretations are also required to classify the non–self-touching behavior of cleaner fish as self-inspection guided by a mirror. Without any training, marked fish spent much time next to the mirror. Whether they looked at themselves was hard to ascertain, but they did orient to the mirror such that they could potentially see the visually marked side of their body and did so more frequently than they did for the unmarked or sham-marked side. Most importantly, the authors argue, the fish showed high rates of self-scraping on a substrate, especially throat-scraping after having been marked on the throat. They did not show this behavior after having received an invisible mark or in the absence of a mirror.

The authors go on to claim that cleaner wrasses exhibit “responses that fulfill the criteria of the mark test.” However, this extraordinary claim hinges on their view that self-scraping, and the way it varies with marks and mirrors, is equivalent to the mark-directed self-exploration with hands or trunks by humans, apes, and elephants, or the mirror-guided self-viewing reported for dolphins. But in the dolphins' case the marked areas were far more variable, as was their behavior in front of a mirror; some behavior was never seen away from it [4,17]. The fish in the study under discussion, in contrast, performed a single stereotypical act after having seen what may have seemed to be another fish carrying an ectoparasite. This makes it hard to be sure that this response constitutes self-exploration, especially because this species is adapted to detect and remove ectoparasites from other fish. True, self-scraping is not a behavior one would expect if these fish interpret their reflection as another individual, but is this enough reason to conclude that they perceive the fish in the mirror as themselves? After all, the most compelling evidence for the latter would be unique behavior never seen without a mirror, whereas self-scraping, or glancing, is a fixed action pattern of many fish. We may need an in-depth study of this particular pattern before we can ascertain what it means when performed in front of a mirror.

One crucial aspect of the mark test by Kohda and colleagues is that the subcutaneously injected elastomer that puts a color mark on the fish is likely to be painful, or at least an irritant. The study controls for this possibility by having sham marks without the color, which indicate that the tactile sensation alone cannot explain the fish's behavior in front of the mirror. But the study does not control for a possible effect of pairing an intense physical sensation with a visual mark. Two recent studies on rhesus macaques illustrate the importance of this multimodality. These monkeys lack MSR if tested with a purely visual mark, but after having received a head implant they use the mirror to groom around the implant. The implant represents a huge abnormal visual stimulus associated with a tactile sensation that is probably quite painful [18]. In another study, rhesus monkeys received food rewards to induce a visual-somatosensory association by projecting painful laser beams onto the monkeys' faces while forcing them to stare at themselves in a mirror. After having thus enhanced the stimulus' salience in thousands of trials, monkeys touched marks wherever they saw them, such as on walls and on other monkeys, including on themselves, during a mirror test involving a dye mark [13].

These studies demonstrate that the combination between a visual mark and a physical irritation helps monkeys make the connection between their own body and the specular image. Because the physical sensation alone or the visual mark alone does not allow them to do so, it is as if these animals need multimodal stimulation to get there. Apes, in contrast, show untrained MSR based on the visual sense alone. Instead of a traditional mirror mark test, monkeys thus appear to pass what could be called a Felt Mark Test [19]. This is also the test applied by Kohda and colleagues, because the marks put on their fish were both visual and somatosensory. For the moment, therefore, my conclusion is that these fish seem to operate at the level of monkeys, not apes.

This is remarkable enough, though, because as opposed to the Big Bang theory of self-awareness, it is more realistic to adopt a gradualist perspective (Fig 3). It seems a gross simplification to lump all animals without MSR into a single cognitive category, from relatively small-brained birds (e.g., a robin’s unabating territorial attacks on its reflection in a window pane) to animals such as cats and dogs, which habituate quickly to their mirror image and learn to ignore it, or monkeys and African Grey parrots, which successfully use a mirror to locate out-of-sight objects [20,21]. Whether pigs can do the same remains unresolved [22,23].

**Fig 3:**
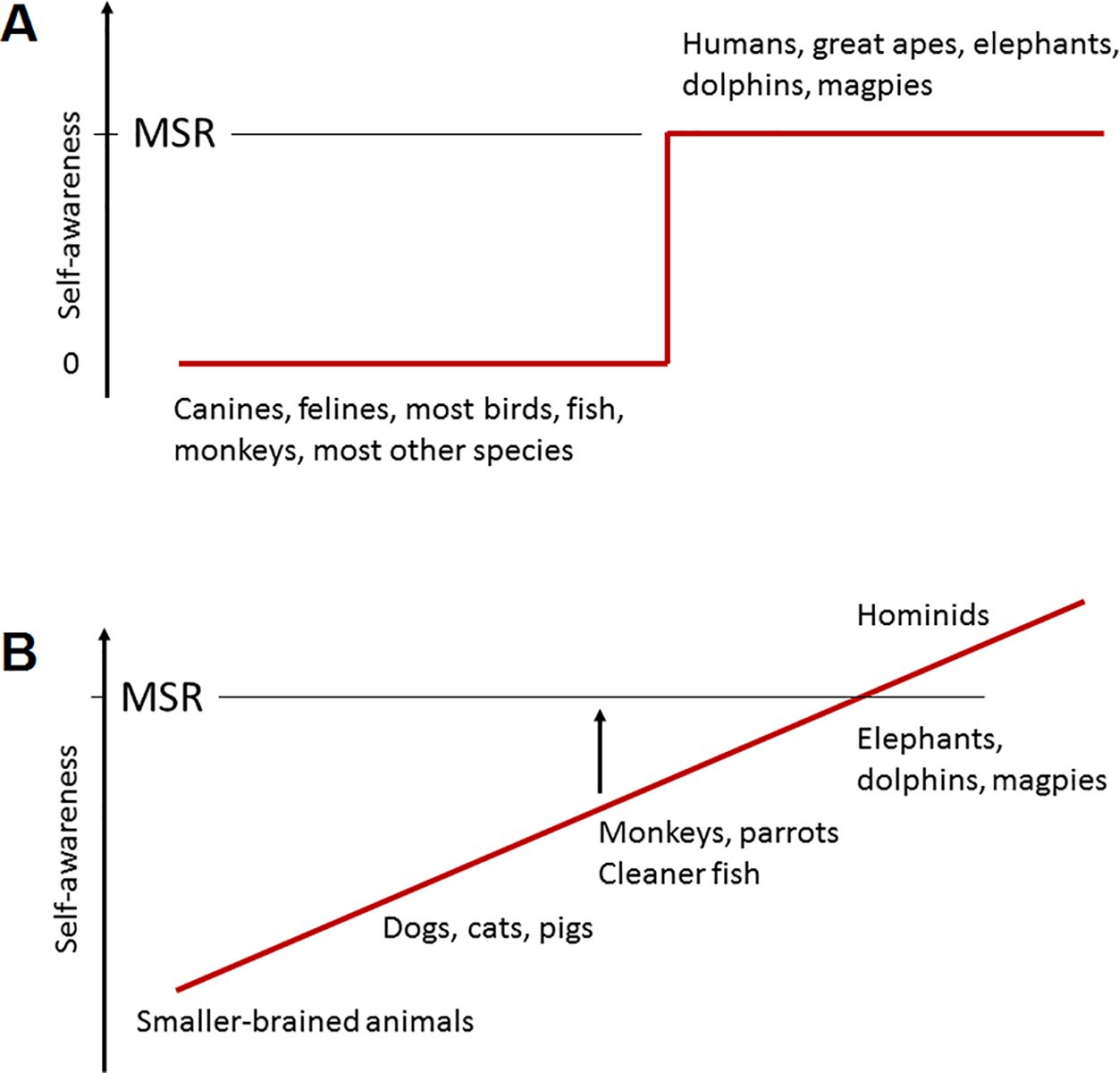
Two different perspectives on the evolution of self-awareness. In the traditional binary model (A), species showing MSR possess a self-concept, whereas all other species do not. The gradualist view (B), in contrast, assigns the highest level of self-awareness to hominids, who spontaneously explore and play with their reflection and care about their appearance, and assigns intermediate or lower levels to other species, but no zero level because all animals need a self-concept. Reactions to mirrors range from permanent confusion about one's reflection to a certain level of understanding of how mirrors operate (e.g., using them as tools) and only brief or no confusion between one's reflection and a stranger. Some species, such as macaques and perhaps cleaner fish, seem to possess this intermediate level and can therefore, with the aid of training and/or multimodal stimulation, be "lifted" (arrow) to a level of mirror understanding closer to MSR. MSR, mirror self-recognition.

It is incorrect to assume, for example, that non-MSR animals merely see an unexpected conspecific in the mirror. This may be true for robins and Siamese fighting fish, but when brown capuchin monkeys were tested facing either a mirror, a familiar monkey, or an unfamiliar monkey, they were remarkably friendly to and interested in their own reflection. Females made about 38 times more eye contact with their mirror image than with a stranger, and males about 11 times. Strangers, in contrast, only induced fear and avoidance. The differences did not seem to reflect learning, at least not during the experiment itself, because they emerged at first exposure [24]. Similarly, the heart rate of macaques confronted with a stranger rises at first, then drops, whereas their heart rate drops right away upon mirror exposure [25]. Some non-MSR species seem closer to mirror understanding than others, therefore. They may not recognize themselves, but they also realize that their reflection is no stranger.

What if self-awareness develops like an onion, building layer upon layer, rather than appearing all at once? Such a model has been proposed for its development in human children, who express curiosity about their reflection well before passing the mirror mark test [26]. Moreover, all animals need a self-concept. A monkey needs to know if a branch can carry his weight before landing on it, or whether he has the strength and skill to win a fight before challenging another individual. Animals need to be aware of the place and affordances of the self in its physical environment as well as the role of the self in their social group [27,28]. Therefore, to explore self-awareness further, we should stop looking at responses to the mirror as the litmus test. There are many other evaluations possible, such as when macaques are able to distinguish a self-controlled cursor on a computer screen from one that moves on its own [29], when chimpanzees find hidden food by watching their own hand move via closed-circuit television [30], when elephants know when their own bodies interfere with performance on a task [31], or when dogs pay more attention to a novel odor added to a sample of their urine than to either uncontaminated urine or the novel odor alone [32]. We need a much larger test battery, including nonvisual tasks, to develop a full understanding of how other species position the self in the world.

The next frontier will be to see whether animals care about how they look in the eyes of others to the point of embellishing themselves, the way we do with makeup, earrings, toupees, and the like. This possibility was first hinted at by observations of a female orangutan at a zoo, who would decorate herself by gathering lettuce leaves from her cage to pile them onto her head while inspecting herself closely in the mirror [33] (Fig 4). Similarly, chimpanzees sometimes adorn themselves by walking around with the skin of monkey prey around their necks or develop a group-wide "fashion" to insert grass into their ears [34,35]. Only with a richer theory of the self and a larger test battery will we be able to determine all of the various levels of self-awareness, including where exactly fish fit in.

**Fig 4:**
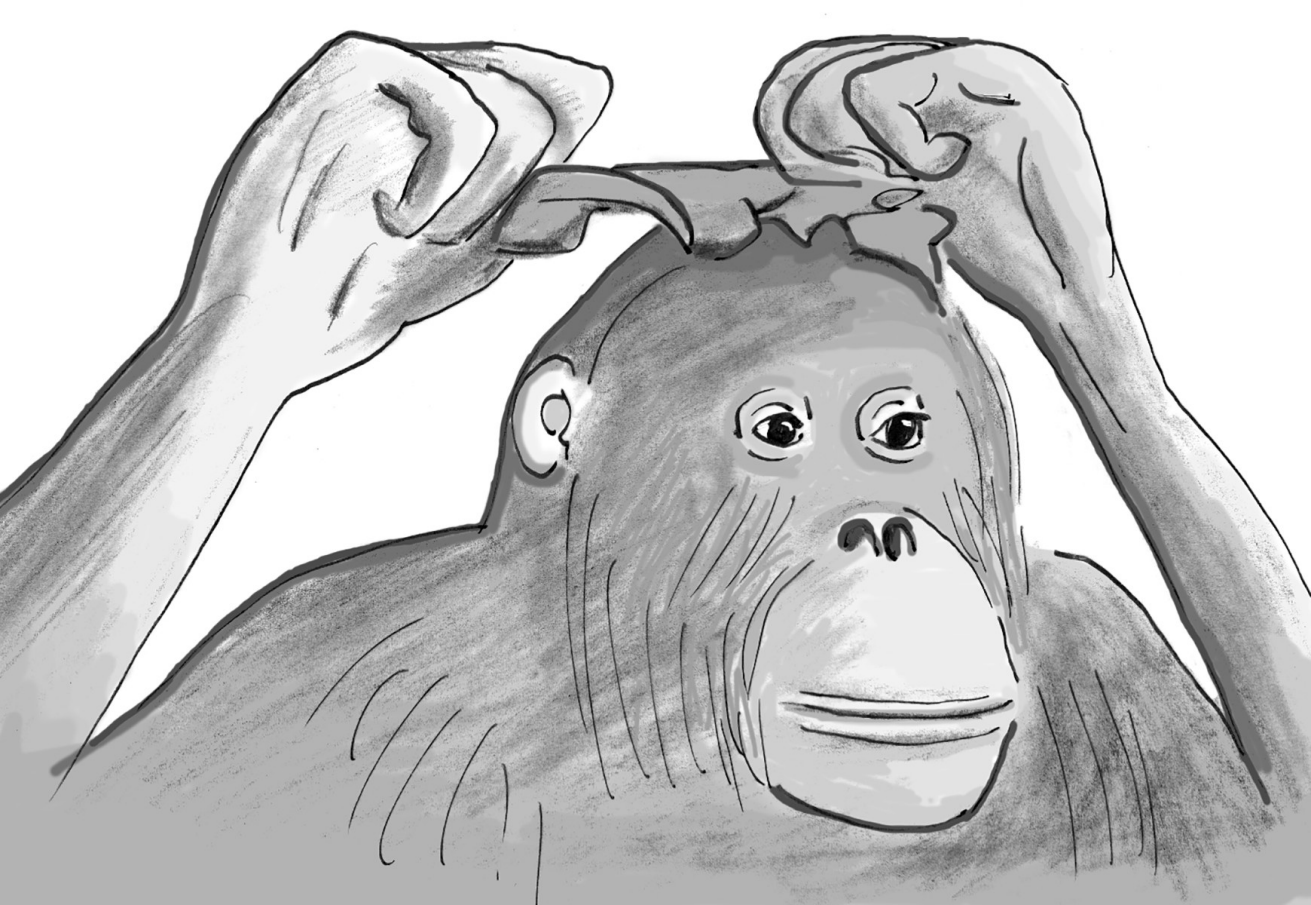
Mirror-guided self-decoration by an ape. Suma, an orangutan at a German zoo, often embellished herself in front of a mirror, such as by putting a leaf of lettuce onto her head like a hat while staring at her reflection. Drawing by Frans de Waal [19] based on [33].

## Abbreviation

MSR: mirror self-recognition
